# A long non-coding RNA expression signature to predict survival of patients with colon adenocarcinoma

**DOI:** 10.18632/oncotarget.21064

**Published:** 2017-09-19

**Authors:** Weinan Xue, Jingwen Li, Fan Wang, Peng Han, Yanlong Liu, Binbin Cui

**Affiliations:** ^1^ Department of Colorectal Surgery, The Affiliated Tumor Hospital of Harbin Medical University, Harbin, 150081, China; ^2^ Department of Epidemiology, School of Public Health, Harbin Medical University, Harbin, 150081, China

**Keywords:** biomarkers, colon adenocarcinoma, expression profiles, long non-coding RNA

## Abstract

Colon cancer is the most common type of gastrointestinal cancer and is still the leading cause of cancer-related mortality worldwide. Long non-coding RNAs (lncRNAs) have been proved to be superior biomarkers in cancer diagnosis and prognosis than miRNAs and protein-coding genes. In the current study, our objective was to detect novel lncRNA biomarkers by analyzing lncRNA expression profiles and clinical data in a large cohort of patients with colon patients from The Cancer Genome Atlas (TCGA). By using Cox regression analysis, we identified two lncRNAs (*SNHG6* and *CTD-2354A18.1*) which could be independent prognostic factors for predicting clinical outcome in colon adenocarcinoma. Then a linear combination of these two lncRNA biomarkers (*SNHG6* and *CTD-2354A18.1*), termed two-lncRNA signature, was developed in the training set as a predictor for OS in patients with colon adenocarcinoma, and validated in the testing set and the entire patient set. This two-lncRNA signature demonstrated significant prognostic performance in both the testing set and the entire patient set which classified the patients into two groups with significantly different OS. The multivariate and stratified analysis suggested that the prognostic value of the two-lncRNA signature was independent of other traditional clinical variables. Functional analysis suggested that these two lncRNA biomarkers might be mainly involved in transcription/translation-related or DNA repair-related biological processes. In summary, our results warrant further studies on these lncRNAs that will improve our understanding of the mechanisms associated with pathogenesis and progression of colon adenocarcinoma.

## INTRODUCTION

Colon cancer is the most common type of gastrointestinal cancer and remains the leading cause of cancer-related mortality worldwide [1]. Colon adenocarcinoma is the most common colon cancer type and accounts for 98% of newly diagnosed colon cancer cases. Surgery combined with other therapeutic options (including cryotherapy, radiofrequency ablation or chemotherapy) is the standard treatment strategy. Despite advances in diagnosis and treatment, the prognosis for patients with metastatic disease remains poor [2]. Hence, the identification of more exact molecular biomarkers for identifying high-risk patient subgroup in survival is evidently needed and would enable clinicians to choose more suitable treatment strategies thus leading to improved clinical outcomes

The advent of full genome sequences and comprehensive analysis of functional elements have suggested that a large portion of the genome can be transcribed and led to the discovery of extensive transcription of large RNA transcripts termed long noncoding RNAs (lncRNAs) [3, 4]. lncRNAs are arbitrarily defined as ncRNA genes larger than 200 bp which clearly distinguishes from small regulatory RNAs [5]. It is now well appreciated that lncRNAs exert a diverse spectrum of regulatory mechanisms in numerous biological processes at the transcriptional level, post-transcriptional level and epigenetic level [6, 7]. Like miRNAs and protein-coding genes, lncRNA also revealed differential expression in carcinogenesis compared to normal tissues, some of which have been identified as a tumor suppressor or oncogenes associated with cancer pathogenesis and development [8–11]. Furthermore, accumulating reports of aberrant lncRNA expression have suggested that lncRNAs may potentially serve as novel independent biomarkers for early diagnosis, prognosis and metastasis prediction in various cancer types [12–18]. Recently, several lncRNA profiling has been done in colorectal cancer and identified novel molecular subtypes and candidate diagnostic and prognostic biomarkers, such as *MALAT1*, *HOTAIR*, *RP11-462C24.1*, *PCAT-1* and so on [19–26]. However, research for lncRNA biomarkers in colon adenocarcinoma is presently in its infancy.

In the current study, our objective was to detect novel lncRNA biomarkers by analyzing lncRNA expression profiles and clinical data in a large cohort of patients with colon patients from The Cancer Genome Atlas (TCGA). Furthermore, we tire to identify a lncRNA-based expression signature to predict patient's clinical outcome. Finally, we performed bioinformatics analysis to infer the possible biological functions of lncRNA signature in colon adenocarcinoma.

## RESULTS

### Identification of prognostic lncRNAs in the training set

To detect the prognostic lncRNAs in colon adenocarcinoma patients, we first performed univariate Cox regression analyses to explore the association between expression levels of lncRNAs and the OS of the patients with colon adenocarcinoma, and identified 14 lncRNAs (*p* < 0.01) that are significantly associated with OS in the training set (Supplementary Table 1). Then we performed multivariate Cox regression analysis for these 14 lncRNAs and found that two of 14 lncRNAs (*SNHG6* and *CTD-2354A18.1*) may be independent prognostic factors (*p* < 0.1) (Table 1). Of them, we found that higher expression level of *SNHG6* was associated with good outcome and a higher expression level of *CTD-2354A18.1* was associated with poor outcome.

**Table 1:** Overall information of two prognostic lncRNAs associated with OS

Ensembl ID	Gene name	Genomic location	*p* value	HR	Coefficient
ENSG00000245910	SNHG6	Chr 8: 66,921,684–66,926,398 (−)	0.008	0.398	−0.921
ENSG00000261780	CTD-2354A18.1	Chr 18: 73,324,941–73,349,878(+)	0.008	2.227	0.8005

### Development of lncRNA expression signature for survival prediction in the training set

**Figure 1:**
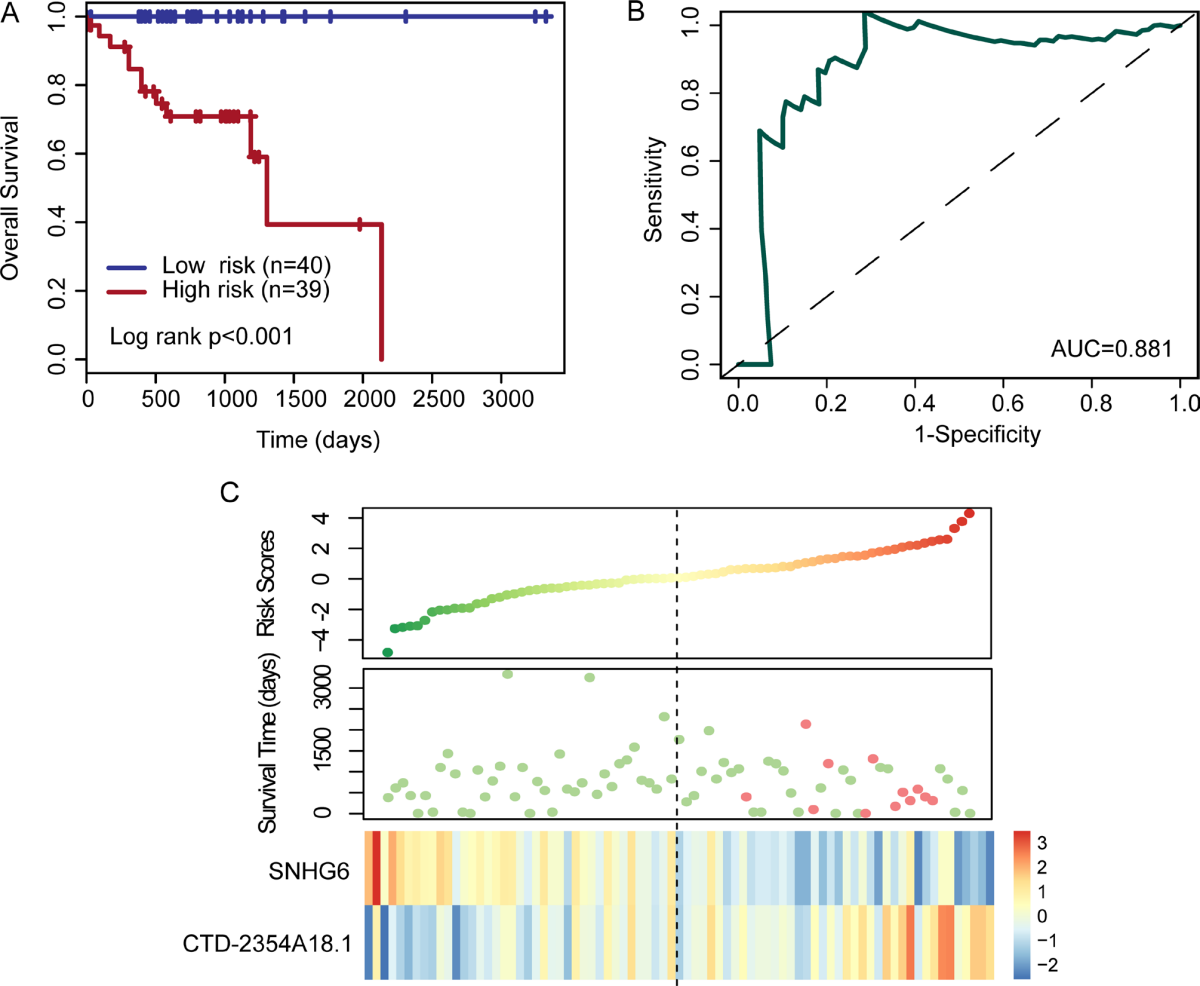
Kaplan–Meier and ROC curves for the two-lncRNA signature in the training set (**A**) Kaplan–Meier curves for high- and low-risk groups obtained from the training set (*n* = 79) divided by the median cutoff point. (**B**) Time-dependent ROC analysis for the two-lncRNA signature at three years of OS. (**C**) Risk score distribution, patients’ survival status and expression heatmap of two lncRNA biomarkers.

Selected two prognostic lncRNAs were fitted in a multivariate Cox regression analysis to obtain their relative coefficient. Then a two-lncRNA expression signature was developed by constructing a mathematical formula using a linear combination of the expression values of two prognostic lncRNAs (*SNHG6* and CTD-2354A18.1) and the multivariate Cox regression coefficient as the weight as follows: LncRNA signature-based risk score = (−1.2023*expression value of *SNHG6*) + (1.106* expression value of *CTD-2354A18.1*). Finally, each of the patients in the training set could get a risk score based on two-lncRNA expression signature and classified into high-risk group and low-risk group using the median risk score of the training set as the risk cutoff. As shown in Figure 1A, patients with low-risk score had a better OS than those with high-risk scores (log rank *p* < 0.001) (Figure 1A). The area under the curve (AUC) of time-dependent ROC curves for the two-lncRNA expression signature was 0.881 at three years of OS (Figure 1B). The hazard ratios of high-risk group versus low-risk group for OS were 2.718 (*p* < 0.001; 95% CI, 1.681–4.397) in the univariate analysis (Table 2). Distribution of the lncRNA risk score, the survival status of the patients and the expression pattern of two prognostic lncRNAs was also shown in Figure 1C. We found that patients in the high-risk group tended to express high level of *CTD-2354A18.1*, whereas patients in the low-risk group tended to express high level of *SNHG6* (Figure 1C).

**Table 2:** Univariate and multivariate Cox regression analysis in each patient set

Variables	Univariate analysis			Multivariate analysis		
	HR	95% CI of HR	*p* value	HR	95% CI of HR	*p* value
**Training set (*n* = 79)**						
Two-lncRNA risk score (High/Low)	2.718	1.681–4.397	< 0.001	4.229	1.835–9.748	0.001
Age	1.093	1.019–1.173	0.013	1.111	1.029–1.200	0.007
Gender (Male/Female)	0.752	0.218–2.597	0.653	3.367	0.737–15.390	0.117
Stage (III,IV/I,II)	1.945	0.624–6.061	0.252	6.746	1.472–30.910	0.014
**Testing set (*n* = 78)**						
Two-lncRNA risk score (High/Low)	2.720	0.984–7.516	0.054	2.762	0.981–7.773	0.054
Age	1.045	0.992–1.100	0.095	1.048	1.002–1.097	0.042
Gender (Male/Female)	0.667	0.240–1.859	0.439	0.418	0.139–1.251	0.119
Stage (III,IV/I,II)	2.982	1.063–8.363	0.038	3.308	1.151–9.508	0.026
**Entire patient set (*n* = 157)**						
Two-lncRNA risk score (High/Low)	5.441	2.196–13.478	< 0.001	4.831	1.947–11.988	0.001
Age	1.063	1.021–1.107	0.003	1.062	1.022–1.103	0.002
Gender (Male/Female)	0.706	0.321–1.552	0.386	0.660	0.299–1.456	0.303
Stage (III,IV/I,II)	2.398	1.131–5.082	0.023	2.539	1.166–5.525	0.019

### Further validation of lncRNA expression signature for survival prediction in the testing set and entire patient set

**Figure 2:**
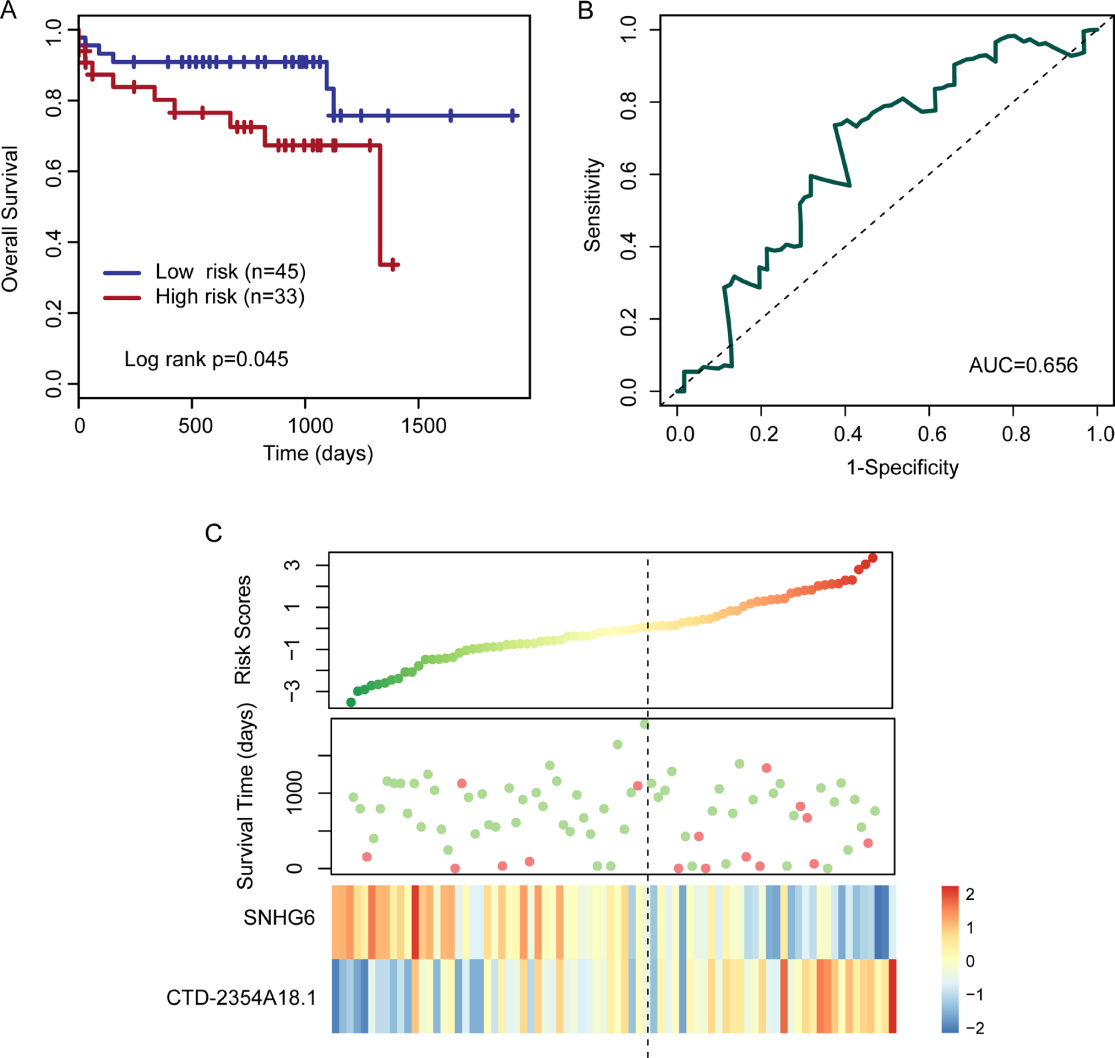
Kaplan–Meier and ROC curves for the two-lncRNA signature in the testing set (**A**) Kaplan–Meier curves for high- and low-risk groups obtained from the testing set (*n* = 78) divided by the median cutoff point. (**B**) Time-dependent ROC analysis for the two-lncRNA signature at three years of OS. (**C**) Risk score distribution, patients’ survival status and expression heatmap of two lncRNA biomarkers.

The two-lncRNA expression signature was then validated for its prognostic value in the testing set of 78 patients. By using the same lncRNA signature-based risk score formula, patients of the testing set were divided into the high-risk group (*n* = 33) and the low-risk group (*n* = 45) the median score of the training set as the cutoff value. In the consistent with the findings from the training set, patients in the high-risk group had significantly shorter median OS than those in the low-risk group (log rank *p* = 0.045) (Figure 2A). The AUC for the two-lncRNA expression signature was 0.656 at three years of OS (Figure 1B). In the univariate analysis, the hazard ratios of high -risk group versus low-risk group for OS were 2.72 (*p* = 0.054; 95% CI, 0.984–7.516) (Table 2). Distribution of the lncRNA risk score, the survival status of the patients and expression pattern of two prognostic lncRNAs was also shown in Figure 2C. We found that the expression pattern of two prognostic lncRNAs in patients with high-risk score or low-risk score is consistent with observations in the training set (Figure 2C).

**Figure 3:**
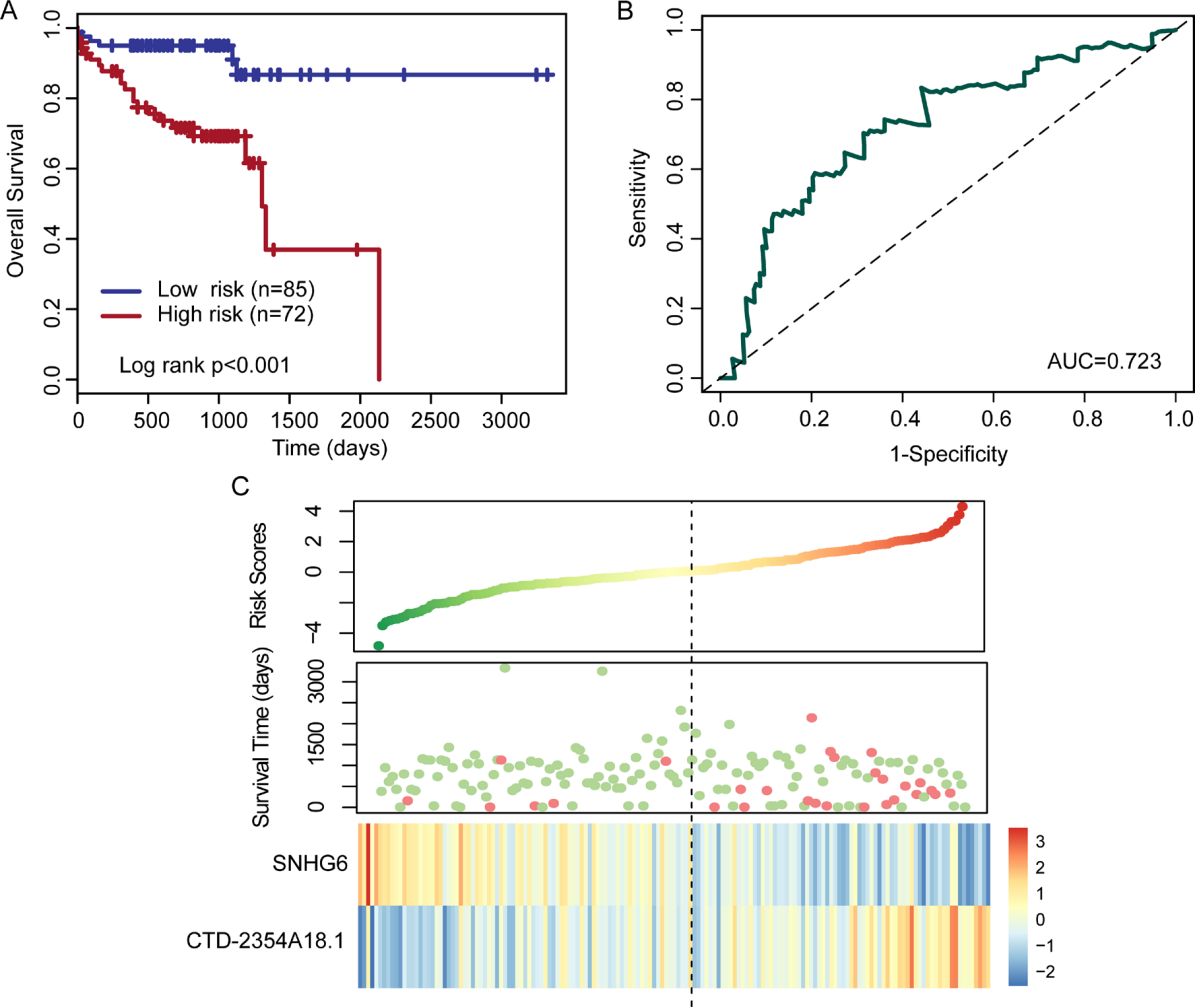
Kaplan–Meier and ROC curves for the two-lncRNA signature in the entire patient set (**A**) Kaplan–Meier curves for high- and low-risk groups obtained from the entire patient set (*n* = 157) divided by the median cutoff point. (**B**) Time-dependent ROC analysis for the two-lncRNA signature at three years of OS. (**C**) Risk score distribution, patients’ survival status and expression heatmap of two lncRNA biomarkers.

We further validated the two-lncRNA expression signature in the entire patient set (combined training set and testing set). With the two-lncRNA expression signature, patients of the entire patient set were divided into a high-risk group (*n* = 72) or a low-risk group (*n* = 85) with significantly different OS (log rank *p* < 0.001), which was similar to those observed in the training set and testing set (Figure 3A). The hazard ratios of high -risk group versus low-risk group for OS were 5.441 (*p* < 0.001; 95% CI, 2.196–13.478) in the univariate analysis (Table 2). The AUC for the two-lncRNA expression signature was 0.723 at three years of OS (Figure 3B). Distribution of the lncRNA risk score, the survival status of the patients and the expression pattern of two prognostic lncRNAs were also shown in Figure 3C.

### Prognostic value of the lncRNA expression signature is independent of clinical variables

To examine whether the prognostic value of the lncRNA expression signature for survival prediction is independent of other clinical variables, we performed multivariate Cox regression analysis with age, gender, stage and lncRNA signature-based risk score as covariables in each of three patient sets. Results from multivariate Cox regression analysis suggested that the lncRNA expression signature, age and stage were significantly correlated with OS of the patients with colon adenocarcinoma in all three patient sets (Table 2).

**Figure 4:**
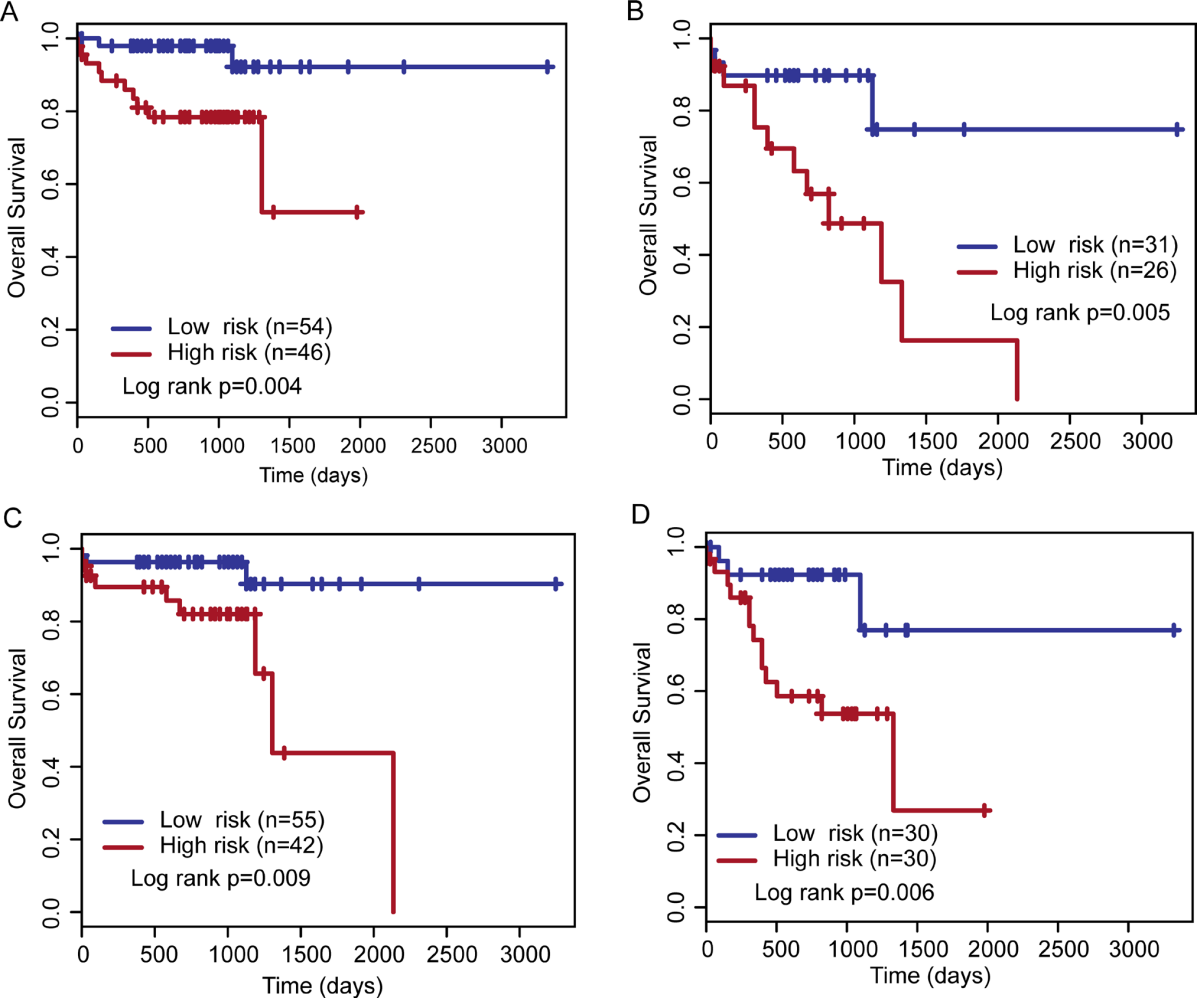
Stratified analysis for age and stage The Kaplan–Meier curves for high- and low-risk groups obtained from younger patients (**A**), elder patients (**B**), early-stage patients (**C**) and advanced-stage patients (**D**).

We next carried out a stratified analysis for age and stage to evaluate whether the lncRNA expression signature could predict OS of patients with the same age and stage features. All patients were stratified into a younger stratum (< 75) or an elder stratum. Results of survival analysis suggested that within each stratum, the two-lncRNA expression signature could further subdivide patients into the high-risk group and low-risk group with significantly different survival time (log rank *p* = 0.004 for younger stratum and log rank *p* = 0.005 for elder stratum) (Figure 4A and 4B). Then all patients were early-stage stratum (I/II) and advanced-stage stratum (III/IV). With the two-lncRNA expression signature, patients with early-stage were classified into the high-risk group (*n* = 42) and low-risk group (*n* = 55). The OS time of the high-risk group patients was significantly shorter than that of low-risk group patients (log rank *p* = 0.009) (Figure 4C). Similar results were observed when patients with advanced-stage stratum were classified as high-risk (*n* = 30) or low-risk (*n* = 30) according to their two-lncRNA expression signature (log rank *p* = 0.006) (Figure 4D).

### Functional enrichment analysis of genes correlated with the prognostic lncRNAs

To investigate the potential functional roles of identified prognostic lncRNAs, we first examined the expression correlation between prognostic lncRNAs and protein-coding genes (PCGs) for all patients and identified 327 PCGs positively or negatively co-expressed with prognostic lncRNAs (top 5%). Then we performed functional enrichment analysis for 327 PCGs positively or negatively co-expressed with prognostic lncRNAs using DAVID web tool. Results of GO analysis suggested that these PCGs correlated with the prognostic lncRNAs were enriched in two functional clusters involved in transcription/translation-related or DNA repair-related biological processes (Table 3). KEGG functional enrichment analysis suggested that PCGs correlated with the prognostic lncRNAs were involved in five biological pathways, including Ribosome, Melanogenesis, Wnt signaling pathway, Glutamatergic synapse and Gastric acid secretion (Table 3). Moreover, 327 PCGs were found to be associated with INFECTION and CANCER disease classes through enrichment analysis in GAD_DISEASE_CLASS (Table 3).

**Table 3:** Enriched functional category in GO, KEGG and disease

GO terms and KEGG pathways	NO. of genes	*p* value
**Functional clusters of GO terms**		
**Cluster 1 (Enrichment Score: 17.97)**		
translational initiation	28	1.4E–21
SRP-dependent cotranslational protein targeting to membrane	24	7.8E–21
viral transcription	25	3.2E–20
nuclear-transcribed mRNA catabolic process, nonsense-mediated decay	25	1.5E–19
rRNA processing	27	2.1E–15
translation	28	1.5E–14
**Cluster 2 (Enrichment Score: 1.18)**		
transcription-coupled nucleotide-excision repair	6	7.7E–3
nucleotide-excision repair, preincision complex assembly	3	8.3E–2
DNA damage response, detection of DNA damage	3	1.2E–1
nucleotide-excision repair, DNA incision, 5’-to lesion	3	1.3E–1
nucleotide-excision repair, DNA incision	3	1.3E–1
**KEGG pathway**		
Ribosome	26	1.3E–18
Melanogenesis	7	1.1E–2
Wnt signaling pathway	8	1.4E–2
Glutamatergic synapse	7	1.9E–2
Gastric acid secretion	5	4.7E–2
**GAD_DISEASE_CLASS**		
INFECTION	51	2.3E–2
CANCER	67	2.9E–2

## DISCUSSION

Colon cancer is a multifactorial disease with etiology encompassing genetic factors, environmental exposures and inflammatory conditions of the digestive tract. Although traditional prognostic and predictive factors for colorectal cancer such as age, tumor stage, surgical margins, number of affected local lymph nodes and tumor grade have produced significant improvements in patient clinical outcome [27], they reveals obvious limitations in distinguishing related cancer risk subgroup that have different clinical outcomes due to the molecular heterogeneity. Therefore, the prognostic potential of molecular markers has been systematically investigated in extensive clinical transcriptome research over the last decade. For example, a well-known gene signature, ColoPrint^®^, was developed to predict disease relapse in patients with early-stage colorectal cancer [28]. Abdul *et al.* identified a 19-gene expression signature as a predictor of survival in colorectal cancer [29]. Another study reported prognostic signature comprising of 113 probe sets (termed CRC-113) to predict prognosis in patients with colorectal cancer [30].

With increasing knowledge in the field of ncRNA research, the development in the identification of molecular markers has been the shift in focus from protein-coding genes to ncRNAs [31, 32]. Some miRNA-based signatures were firstly identified in recent studies [33, 34]. Recently, lncRNAs have been discovered as a key component of genome regulatory network and contribute to the hallmark of cancer [5, 35]. Accumulating evidence showed that lncRNA displays restricted tissue-specific and cancer-specific expression patterns distinguishing from miRNAs and protein-coding genes [8]. Moreover, tissue-specific and cancer-specific lncRNA expression was detectable and stable in body fluids, sputum and urine of cancer patients [8]. This specificity represents potentials of lncRNAs as superior biomarkers in cancer diagnosis and prognosis than miRNAs and protein-coding genes [36–39]. Several groups have initially investigated the prognostic value of lncRNA in colon cancers. Chen *et al.* used unsupervised consensus clustering method to identify five distinct molecular subtypes of CRC with different clinical outcome [19]. In one lncRNA profiling study based on GEO data, a lncRNA expression signature containing six lncRNA was first identified to improve prognosis prediction of colorectal cancer [40]. A recent study confirmed eight novel lncRNAs as associated with the progression of colon cancer [26]. However, it should be noted that there was little overlap between lncRNAs identified as biomarkers in colorectal cancer in previous studies, indicating that seeking lncRNA biomarkers of colorectal cancer is presently in its infancy and the further comprehensive investigation is necessary.

In this study, we performed integrative analysis for lncRNA expression profiles and clinical data in 157 patients with colon adenocarcinoma from TCGA to access the prognostic value of lncRNA expression for OS in patients with colon adenocarcinoma. We first subjected the lncRNA expression profiles of 79 patients in the training set to Cox proportional regression analysis for evaluating the relationship between lncRNA expression and clinical outcome, and identified two lncRNAs (*SNHG6* and *CTD-2354A18.1*) which could be considered as independent prognostic factors for predicting clinical outcome in colon adenocarcinoma. More important, using a sample-splitting approach, a linear combination of these two lncRNA biomarkers (*SNHG6* and *CTD-2354A18.1*), termed two-lncRNA signature, was developed in the training set as a predictor for OS in patients with colon adenocarcinoma, and validated in the testing set and entire patient set. This two-lncRNA signature demonstrated significant prognostic performance in both the testing set and entire patient set which classified the patients into two groups with significantly different OS. Further analysis revealed that the prognostic value of the two-lncRNA signature was independent of other traditional clinical variables, such as age, gender, stage. Moreover, in the stratified analysis, the two-lncRNA signature showed prognostic value both in early-stage and advanced-stage patients, and both in younger and elder patients. These suggested that the two-lncRNA signature is an independent prognostic predictor in colon adenocarcinoma.

The two-lncRNA signature identified in this study included one lncRNAs (*SNHG6*) that was protective and another lncRNA (*CTD-2354A18.1*) that was risky with respect to their association between their expression and patients’ survival. In the literature, lncRNA *SNHG6* has been reported to be differentially expressed in hepatocellular carcinoma (HCC) and may be a potential biomarker for patients with HCC [41]. Yang's study found that *SNHG6* is overexpressed in gastric cancer tissues and cell lines measured by quantitative real-time polymerase chain reaction (qRT-PCR) and might serve as a candidate prognostic biomarker for gastric cancer patients [42]. Another lncRNA biomarker, *CTD-2354A18.1*, has also been shown to differentially expressed in gastric cancer tissue and normal tissue and may play a key role in the pathogenesis of gastric cancer [43]. However, the functional roles of these two lncRNA biomarkers in colon adenocarcinoma still remain to be explored. We infer potential functional roles of these two lncRNA biomarkers in colon adenocarcinoma performed by GO and KEGG pathway functional enrichment analysis, and found that these two lncRNA biomarkers may be mainly involved in transcription/translation-related or DNA repair-related biological processes, which is consistent with previous study [41].

In conclusion, our study identified a novel lncRNA expression signature comprising two lncRNAs (*SNHG6* and *CTD-2354A18.1*), which can be used as an independent prognostic marker of OS for patients with colon adenocarcinoma. More importantly, this lncRNA signature could provide additional prognostic information beyond clinicopathological factors. Our results warrant further studies on these lncRNAs that will improve our understanding of the mechanisms associated with pathogenesis and progression of colon adenocarcinoma.

## MATERIALS AND METHODS

### Patient dataset

Colon adenocarcinoma patients and corresponding clinical data used in this study were obtained from were obtained from The Cancer Genome Atlas (TCGA) data portal (https://cancergenome.nih.gov/). Then downloaded clinical data were matched to the lncRNA expression profile. Therefore, some patients without clinical information or lncRNA expression profile were excluded. Finally, a total of 157 colon adenocarcinoma patients were retained for further analysis. In this study, 157 colon adenocarcinoma patients were randomly assigned to a training set (*n* = 79) and a testing set (*n* = 78). The training set was used to detect prognostic lncRNA. The detailed clinical characteristics of the colon adenocarcinoma patients are summarized in Table 4.

**Table 4:** Clinical characteristics of colon adenocarcinoma patients

Variables		Training set (*n* = 79)	Testing set (*n* = 78)	Entire TCGA set (*n* = 157)
Survival status, *n* (%)	Alive	67	62	129
	Dead	12	16	28
Age,years, *n* (%)	> 60	59	62	121
	<= 60	20	16	36
Gender, *n* (%)	Female	45	40	85
	Male	34	38	72
Stage, *n* (%)	Stage I/II	51	46	97
	Stage III/IV	28	32	60

### LncRNA expression data procession

Expression levels of 12,727 lncRNA genes from 157 colon adenocarcinoma patients were downloaded from the TANRIC(The Atlas of ncRNA in Cancer, http://bioinformatics.mdanderson.org/) (version 1.0.6) which is an open-access resource for expression profiles of lncRNAs in large patient cohorts of 20 cancer types including TCGA [44]. Briefly, 12,727 lncRNAs was obtained by examining the overlapping between lncRNA exons and any known coding genes based on the annotations of GENCODE and RefGene. Then, expression levels of 12,727 lncRNAs were quantified using reads per kilobase per million mapped reads (RPKM). Due to the very low expression level of lncRNAs Colon adenocarcinoma patients, we filtered out lncRNAs with expression value of 0 in more than 30% samples and obtained 1296 lncRNAs for further analysis. The expressive value of 1296 lncRNAs was log transformed using log2 (RPKM+0.001) and z-score normalized.

### Statistical analysis

Univariate and multivariate Cox regression analysis was first performed to evaluate the association between the expression levels of lncRNAs and the OS of patients with colon adenocarcinoma and to identify prognostic lncRNAs. A lncRNA expression signature was constructed by a linear combination of the expression values of prognostic lncRNAs and the multivariate Cox regression coefficient as the weight. By using the median risk score as the cutoff point, patients can be classified into high-risk and low-risk groups. The Kaplan-Meier curves and log rank test were performed to assess survival differences between the low-risk and high-risk groups using the R package “survival”. Multivariate Cox regression analysis and data stratification analysis was performed to test whether the lncRNA expression signature was independent of clinical features. Time-dependent ROC curves were used to compare the sensitivity and specificity of the three-survival prediction based on the lncRNA expression signature using the R package “survivalROC” [45]. All analyses were performed using the R/BioConductor (version 3.0.2).

### Functional enrichment analysis

Functional enrichment analysis was performed for co-expressed protein-coding RNAs in GO, KEGG and GAD_DISEASE_CLASS using The Database for Annotation, Visualization and Integrated Discovery (DAVID) v6.8 (https://david.ncifcrf.gov/) [46].

## SUPPLEMENTARY MATERIALS TABLE


