# Supplementary material for: A long non-coding RNA expression signature to predict survival of patients with colon adenocarcinoma
Source: Oncotarget. 2017 Sep 19;8(60):101298–308. doi: 10.18632/oncotarget.21064 (PMC5731875; doi:10.18632/oncotarget.21064)
Supplement: Supplementary file 1 [file oncotarget-08-101298-s001.pdf]

A long non-coding RNA expression signature to predict survival of patients with colon adenocarcinoma

SUPPLEMENTARY MATERIALS

Supplementary Table 1: Overview of lncRNAs significantly associated with OS in the training set

	Coefficient	*p*-value	HR	lower .95	upper .95
ENSG00000174365.15	−0.7337	0.0086	0.4801	0.2777	0.8301
ENSG00000174403.11	−0.9853	0.0022	0.3733	0.1985	0.7022
ENSG00000177410.8	−0.8053	0.002	0.4469	0.2679	0.7457
ENSG00000177822.3	0.6529	0.0068	1.9211	1.1974	3.0821
ENSG00000178947.8	−0.9282	0.0092	0.3953	0.1966	0.7946
ENSG00000227619.1	−0.8302	0.0018	0.436	0.2591	0.7337
ENSG00000229891.1	−0.8406	0.0024	0.4315	0.2505	0.743
ENSG00000230487.3	−0.928	0.0041	0.3953	0.2096	0.7455
ENSG00000233559.1	−0.8295	0.0084	0.4363	0.2353	0.8088
ENSG00000234390.4	−0.7629	0.0055	0.4663	0.2722	0.7988
ENSG00000245910.4	−0.921	0.0077	0.3981	0.2022	0.7838
ENSG00000255234.1	0.717	0.006	2.0483	1.2277	3.4172
ENSG00000261780.2	0.8005	0.0077	2.2267	1.2361	4.011
ENSG00000273213.1	1.1831	0.0084	3.2645	1.354	7.8711
